# Comparison of the Effect of Multiple Dietary Supplements on the Quality of Sperm Parameters in Elite and Amateur Soccer Players

**DOI:** 10.1155/jnme/2952412

**Published:** 2025-06-16

**Authors:** Saeid Rostami, Bijan Rajaeian, Nooshin Rajaeian

**Affiliations:** ^1^Department of Exercise Physiology, Ragheb Esfahani Higher Education Institute, Isfahan, Iran; ^2^Department of Motor Behaviour, Islamic Azad University, Shahinshahr Branch, Shahinshahr, Isfahan, Iran; ^3^Department of Kinesiology, Indiana University, Bloomington, Indiana, USA

**Keywords:** football, morphology, motility, sperm, supplement, volume

## Abstract

**Background:** Sperm DNA integrity is a key factor in male fertility and the health of future generations. Physical activities such as football can increase oxidative stress, potentially compromising sperm quality. Dietary supplements targeting oxidative balance and the one-carbon metabolic cycle have shown promise in improving sperm parameters.

**Objective:** This randomized, double-blind clinical trial investigated the effect of multiple dietary supplements (Condensyl and Nurilia) on sperm parameters in elite and amateur football players in Isfahan.

**Method:** Eighty male football players (40 elite and 40 amateur), each with more than two years of experience and abnormal semen parameters, were randomly assigned to supplement or placebo groups using a computer-generated list. Subjects were evaluated for sperm parameters and then treated daily for 3 months with two tablets of multiple diet supplements. For 3 months, subjects received two 600 mg tablets daily (Condensyl, Parthenogen SAGL, Lugano, Switzerland, and Nurilia SARL, Lyon, France). The placebo group also received two 500 mg capsules containing starch daily (Nader Isfahan Limited Liability Company and received the registration number 14906). Semen samples were collected in two stages before and after 3 months of drug use with 4 days of sexual abstinence. Half an hour after sample collection and liquefaction, parameter analysis (concentration, motility, and morphology) was performed. Comparison of sperm parameters before and after drug intervention was performed using SPSS software Version 24 and the Shapiro–Wilk test, followed by multivariate analysis of variance. A significance level of 0.05 was considered.

**Results:** Baseline comparisons revealed significant group differences in motility (*p*=0.024) and morphology (*p*=0.008). Sperm concentration increased in both elite and amateur players receiving supplements, though the change was not statistically significant (*p* > 0.05). However, significant improvements were observed in sperm motility (*p*=0.014 and *p*=0.02) and morphology (*p*=0.01) following supplementation.

**Conclusion:** Supplementation with Condensyl and Nurilia, which include key vitamins and antioxidants supporting the one-carbon cycle, significantly improves sperm motility and morphology in male football players. These findings support the use of such supplements as a supportive therapy in the management of male infertility.

## 1. Introduction

Sperm DNA integrity is one of the essential factors that not only affect developmental competency but also ensure the health of future generations. Recent clinical studies suggest that approximately 60% of men referred to assisted reproductive centers and about 80% of idiopathic infertile men have severe or moderate damage to sperm DNA [1, 2]. One of the most important causes of sperm DNA damage is the production of excessive amounts of reactive oxygen species (ROS), a state called oxidative stress [2]. It is also of note that reductive stress can also result in the production of ROS. Oxidative stress not only affects sperm DNA integrity but also reduces the fertilization potential, possibly through lipid peroxidation which reduces sperm membrane flexibility and thereby sperm motility and its ability to penetrate the oocyte [3]. Increased oxidative stress in the semen of infertile men indicates that oxidative stress plays an important role in structural disorders and functional capacities of sperm through various mechanisms.

Recent studies have shown that muscle contractions, especially exercise, are associated with an increase in the production in ROS in the muscles [2] and 2%–5% of oxygen consumption in muscle mitochondria during oxidative phosphorylation, aerobic activity, is converted to superoxide (O2) as one form of ROS [3]. Oxidative stress refers to the imbalance between oxidants or ROS with antioxidants. Under these conditions the weight changes in favor of oxidants or ROS which is associated with oxidative damage and acceleration of harmful pathological pathways [4]. This condition is also seen during or hours after intense muscle contractions because under these conditions oxygen consumption sometimes increases up to 20 times, which is accompanied by an increase in the production of superoxide [5].

The physiological level of ROS is very important for the vital functions of sperm, including capacitation, acrosome reaction, and fertilization with ovules [6]. If the level of ROS increases compared with the physiological level, it is naturally inactivated by antioxidants in semen plasma. However, when the ROS level exceeds the antioxidant capacity of the reproductive system, it induces damage to the sperm [7, 8]. Research findings show that oxidative stress, like what occurs in sports such as football, is inversely related to number, motility, and fertilization capacity [9–11], which can lead to male-related infertility [12]. Sperm DNA integrity is a critical factor influencing fertility and the health of offspring. Up to 60% of men referred to fertility centers and about 80% of idiopathic infertile men show significant sperm DNA damage. One major contributing factor is oxidative stress caused by excessive ROS. Physical exercise, particularly in sports like football, has been associated with increased oxidative stress, potentially impacting male fertility. Studies have demonstrated that endurance exercises elevate ROS production due to increased oxygen consumption in muscle mitochondria. Although ROS at physiological levels are necessary for sperm function (capacitation and acrosome reaction), excessive levels cause lipid peroxidation, reduced motility, and DNA damage. Some studies have indicated adverse effects of professional football on sperm quality due to sustained oxidative stress [9, 13].

One of the most widely used therapies to reduce ROS is the use of antioxidants. The low cost and relatively low risk of this method have multiplied its effectiveness. On the other hand, there are concerns about excessive reduction of ROS levels because its excessive reduction has a significant effect on vital processes such as sperm chromatin condensation due to disulfide bridges between SH groups of cysteine-enriched protamine [14] Antioxidants are widely used in male infertility therapy, but over-supplementation may disrupt sperm chromatin structure. Giustarini et al. (2008) showed that high-dose vitamin C (≥ 1000 mg/day) might interfere with disulfide bond formation in sperm nuclei. Hence, a balanced supplement targeting the one-carbon cycle is proposed to maintain redox homeostasis while supporting spermatogenesis. In this regard, a study stated that the treatment of infertile men with antioxidant vitamin C leads to adverse effects on sperm nucleus condensation [15] Therefore, the need for a new approach based on body homeostasis to reduce undesirable performance and improve optimal antioxidant performance seems necessary.

The amino acid homocysteine (hcy) is involved in two important metabolic pathways: the glutathione (GSH) synthesis pathway, which is responsible for regulating oxidation–reduction equilibrium, and the transmethylation pathway through one carbon cycle, which regulates gene expression and cell growth. On the other hand, excessive increase of this substance can have negative effects on the quality of spermatogenesis and its accumulation in ejaculate fluid affects fertility. The one carbon pathway is known as an important pathway in which the occurrence of defects increases the level of GSH and increases free hcy and the effect on the methylation process. Therefore, dietary supplements that improve the performance of this pathway strengthen the antioxidants capacity and at the same time, while protecting tissue growth, lead to better sperm differentiation and maturation [16]. It is good to indicate that the one-carbon cycle if compose of 3 cycles, the one-carbon cycle and the trans-sulfation pathway should contain a wide range of B vitamins and folic acid to perform the process of recovery of hcy and zinc as an essential cofactor. On the other hand, it should contain N-acetylcysteine (NAC) as the only oral precursor with strong absorption for GSH synthesis that improves sperm parameters and reduces oxidative stress, which is easily converted to cysteine inside the cell [17]. Multiple dietary supplements have been introduced as a strong supplement supporting the single carbon cycle to treat, improving male fertility, which along with these supplements contains vitamin E, figs, beta-alanine and quercetin of natural origin, which protects the cell membrane against peroxidation. In football players, due to its aerobic and endurance nature, production of ROS increases, which in turn affect sperm parameters and quality and the fertility and quality of life in these athletes. So, the aim of this study was to investigate the effect of one carbon cycle supplementation multiple dietary supplements on the quality of sperm parameters of elite and amateur football players of Isfahan.

The one-carbon cycle involves methionine metabolism and methylation processes essential for DNA synthesis, cell division, and differentiation. Nutritional support using B-complex vitamins, folate, zinc, and NAC can modulate this cycle. NAC, a potent antioxidant precursor to GSH, improves sperm quality through intracellular redox balance. Therefore, the present study aimed to investigate the effect of a comprehensive supplement targeting the one-carbon cycle on sperm parameters in elite and amateur football players from Isfahan.

## 2. Materials and Methods

### 2.1. Study Design

#### 2.1.1. Study Design and Participants

This randomized, double-blind clinical trial which was approved by the Research Ethics Committee of Islamic Azad University (IR.KHUISF.REC.1400.260) and the clinical trial registration system (IRCT20211103052956N1) and the consent form for participation in the study was completed by all subjects.

**Figure 1:**
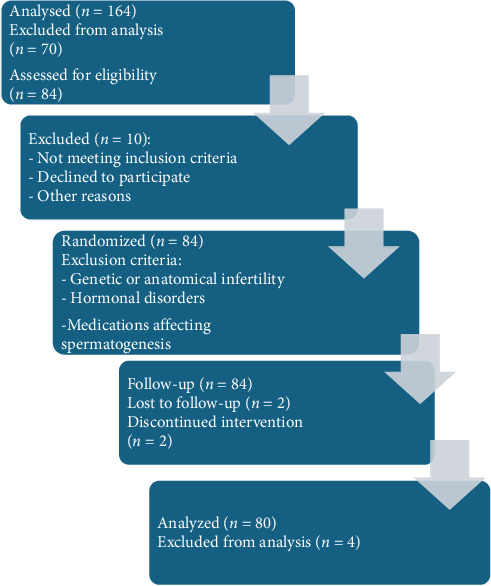
Study design.

The study followed CONSORT 2020 guidelines. A flowchart of the study design is presented in Figure 1. The study was conducted between March and October 2023. Participants included 80 male football players aged 20–35 years, 40 elite (national league players with > 2 years' experience) and 40 amateur (provincial players with > 2 years' experience), with abnormal semen parameters (volume < 1.4 mL, total count < 39 million, and motility < 42%).

#### 2.1.2. Randomization and Blinding

Participants were randomized using a computer-generated sequence into four groups (elite–supplement, elite–placebo, amateur–supplement, and amateur–placebo). Both participants and evaluators were blinded to group allocation.

#### 2.1.3. Intervention

The supplement group received two daily 600 mg tablets of Condensyl/Nurilia containing NAC, vitamins B2, B3, B6, B9, and B12, zinc, vitamin E, and natural antioxidants (e.g., quercetin and beta-alanine). The placebo group received starch-based capsules (500 mg) with identical appearance.

#### 2.1.4. Sample Collection and Analysis

Semen samples were collected after 4 days of abstinence. Volume was measured by digital scale. Sperm concentration and motility were assessed using light microscopy and CASA software (World Health Organization [WHO], 2021). Morphology was evaluated by Diff–Quick staining.

### 2.2. Participants

Participants were collected from among the community of Iranian soccer players in two professional levels (players of the Premier League and with an experience of more than two years in this league) and amateurs (players in the Premier League of Isfahan province with experience of more than two years in this level of Competitions); 80 persons from each of the professional and amateur groups were selected in an accessible and targeted manner, and 40 amateur and 40 professional players were randomly divided into placebo or complementary groups. All participants had abnormal semen parameters, volume was less than 1.4 mL, total count was less than 39 million, and motility was less than 42% (WHO, 2021). After completing the relevant forms and recording the initial information, the subjects were evaluated for sperm parameters and then treated daily for 3 months with two tablets of multiple diet supplements; then, after the end of the diet, they were evaluated again. Sperm parameters were analyzed.

### 2.3. Intervention

For 3 months, subjects received two 600 mg tablets daily (Condensyl, Parthenogen SAGL, Lugano, Switzerland, and Nurilia SARL, Lyon, France) [18, 19]. The placebo group also received two 500 mg capsules containing starch daily (Nader Isfahan Limited Liability Company and received the registration number 14906). Semen samples were collected in two stages before and after 3 months of drug use with 4 days of contraception (sperm quality parameters in the age group < 35 years are the best after 3-4 days of abstinence, while ages > 36 years have better sperm quality parameters after 5-6 days of abstinence) [20]. Half an hour after sample collection and liquefaction, parameter analysis (concentration, motility, and morphology) was performed. The exact volume of the semen sample was measured by a digital scale and the sperm count was performed by a sperm counter slide using a light microscope so that the average numbers were reported during the column count. Sperm motility was assessed according to WHO-2010 criteria by CASA software. Motility included three types: progressive, nonprogressive motility, and immobile sperm. In the present study, motility was reported as the percentage of progressive and nonprogressive motility. After semen analysis, sperm morphology was assessed by Diff–Quick staining using light microscopy and CASA software according to the WHO standard [21]. To evaluate the morphology in this method, the sperm sample was washed once or twice with physiological serum and after preparing and drying the smear at room temperature, the slides were placed in three solutions of methanol, eosin, and thiazine for 30 s in each step, respectively. In each slide, about 200 sperms were morphologically examined using CASA software. In this criterion, natural sperms are considered that have an oval head shape with a smooth environment and the acrosome covers about 40%–70% of the sperm head and also the neck and tail area of the sperm are normal.

### 2.4. Statistical Method

Statistical analysis was performed using descriptive analysis of mean standard error of all standard research parameters. Comparison of sperm parameters before and after drug intervention was performed using SPSS software Version 24, the Shapiro–Wilk test, and multivariate analysis of variance, and a significance level of 0.05 was considered.

## 3. Results

The results are illustrated in two tables. There was no significant difference in baseline levels of subjects' primary sperm parameters (*p* > 0.05). According to a multivariate analysis of variance, sperm concentration increased in both elite and amateur groups with supplementation, but this difference was not statistically significant (*p* > 0.05). Sperm motility increased significantly after receiving the supplement in both elite and amateur groups (*p*=0.014 and *p*=0.02, respectively). The results also showed that the percentage of abnormal morphology after receiving the oral supplement quarter decreased significantly compared to before; in other words, the percentage of sperm with normal morphology increased (*p*=0.01). Also, no significant difference was observed between the sample sizes before and after supplementation (*p*=0.07) (Table 1).

Baseline characteristics were comparable in age and semen volume. However, preintervention differences were observed in motility (*p*=0.024) and morphology (*p*=0.008). These differences were adjusted during analysis (Table 2).

## 4. Discussion

Antioxidant therapy is a treatment for people with low-quality sperm parameters or high levels of oxidative stress. Antioxidants are substances that break the chain of oxidative reactions and, as a result, reduce oxidative stress. The low cost and low risk of these substances are desirable for infertility patients and physicians. Athletes active in endurance disciplines may also experience infertility due to high levels of oxidative stress, and the use of these substances can be very effective. Numerous studies have been conducted in this field that has left contradictory results. The results of the present study showed that with daily consumption of two multiple diet supplements for 3 months, the normal morphology of sperm is significantly improved compared with before or compared with the placebo group, improving sperm morphology by this type of supplement. Similar to previous studies on different antioxidants such as L-carnitine along with coenzyme Q-10, vitamin E, vitamin C, pentoxifylline, and NAC, sperm morphology after receiving such supplements significantly improved [1, 21–26].

Past research has shown that sperm are protected by enzymatic or nonenzymatic antioxidants in semen plasma and prevent oxidative damage. By reducing this ability to use dietary supplements that can reduce oxidative stress and increase sperm health, it will be effective in improving male infertility. The process of oxidative damage in endurance sports and professional athletes has been reported in abundance, and this issue will cause infertility in this type of athletes [26–29].

This trial confirmed the efficacy of multiple dietary supplements in enhancing sperm motility and morphology among football players. These findings are consistent with previous studies showing benefits of antioxidants (e.g., L-carnitine, coenzyme Q10, vitamin E, and selenium) on sperm function. However, inconsistent results regarding Condensyl have been reported; some studies indicate limited effects in men with severe oligozoospermia. Dose-dependent effects of supplement components were not evaluated and should be addressed in future trials. The precise impact of individual ingredients and their synergistic interactions remain to be clarified. Despite baseline differences, multivariate analysis was adjusted for confounders. The trial was limited by the relatively short duration and lack of follow-up on pregnancy rates.

Multiple dietary supplements have the potential to boost the immune system and sperm maturation process, including monocarbon nutritional interventions. B vitamins are cofactors for the main metabolic enzymes of sperm and their amount depends on daily absorption. The combination of zinc and vitamin B9 (folic acid) has a positive effect on sperm nuclei and acrosome [30]. Folate, along with hcy and methionine, is effective in normal sperm maturation and chromatin density and protects sperm from ROS [31]. hcy is recovered by a single carbon cycle, which requires the proper functioning of enzymes. This oral supplement contains B vitamins and important cofactors of the single carbon cycle [30]. On the other hand, water-soluble NAC is easily absorbed and with its antioxidant properties increasing the level of sperm parameters. Vitamin E along with zinc metal increases sperm motility and fertility potential [32]. Quercetin and beta-alanine inhibit the process of lipid peroxidation by reducing the oxidative pressure [14].

## 5. Conclusions

Therefore, the correctness of choosing this type of dietary supplement has been confirmed in previous studies and has a significant effect on balancing the redox process. Considering that the supplement used in the present study included all the vitamins and essential components of the single carbon cycle, improving the quality of sperm parameters in male athletes was one of the objectives of the present study. Considering the analysis of sperm parameters in order to diagnose fertility in men, especially athletes, according to the results of the present study, it can be concluded that multiple dietary supplements improve these characteristics. Supplementation with Condensyl and Nurilia significantly improves sperm motility and morphology in elite and amateur football players. These findings support their use as a supportive therapy in athletes with infertility.

## Figures and Tables

**Table 1:** Changes in research variables in different groups (in millions per cc or percentage of sperm).

Variable	Group	Elite + supplement	Elite + placebo	Elite + supplement	Amateur + placebo	Between groups significance
Volume	Pretest	3/64 ± 0/67	3/94 ± 0/52	3/88 ± 0/74	3/49 ± 0/51	0.619
	Posttest	3/88 ± 0/57	3/73 ± 0/91	3/84 ± 0/74	3/71 ± 0/92	
	Intergroup significance	0/51	0/64	0/064	0/228	

Morphology	Pretest	98/75 ± 1/65	97/67 ± 1/58	97/48 ± 1/81	99/72 ± 1/31	0/024^∗^
	Posttest	92/41 ± 2/53	97/81 ± 1/23	94/69 ± 1/73	98/86 ± 1/47	
	Intergroup significance	0/016^∗^	0/128	0/021^∗^	0/071	

Motility	Pretest	36/9 ± 5/35	35/9 ± 3/22	35/4 ± 2/45	34/2 ± 7/51	0/008^∗^
	Posttest	45/3 ± 4/21	36/2 ± 1/17	42/8 ± 6/51	35/5 ± 3/48	
	Intergroup significance	0/001^∗^	0/73	0/003^∗^	0/404	

Density	Pretest	45/14 ± 2/3	48/5 ± 2/7	46/1 ± 2/5	46/3 ± 2/4	0/619
	Posttest	47/6 ± 1/8	47/1 ± 1/9	47/4 ± 2/6	46/1 ± 1/2	
	Intergroup significance	0/517	0/061	0/074	0/093	

^∗^Significance level.

**Table 2:** Significant improvements were observed postintervention in motility (*p*=0.014, 0.02) and morphology (*p*=0.01) in both supplement groups.

Group	*N*	Volume (mL)	Motility (%)	Morphology (%)	Concentration (mil/mL)
Elite + supplement	20	3.88 ± 0.74	42.8 ± 6.5	94.7 ± 1.7	47.4 ± 2.6
Elite + placebo	20	3.94 ± 0.52	36.2 ± 1.2	97.8 ± 1.2	47.1 ± 1.9
Amateur + supplement	20	3.64 ± 0.67	45.3 ± 4.2	92.4 ± 2.5	47.6 ± 1.8
Amateur + placebo	20	3.49 ± 0.51	35.5 ± 3.4	98.9 ± 1.5	46.1 ± 1.2

## Data Availability

The data that support the findings of this study are available from the corresponding author upon reasonable request.
